# Expression of some selected cytokeratins and Ki67 protein in prostatic tumor: can these be used as tumor markers

**DOI:** 10.11604/pamj.2015.20.46.3926

**Published:** 2015-01-16

**Authors:** James Olayiwola Adisa, Ejike Chukwudi Egbujo, Babangida Ibrahim, Bukar Musa, Jonathan Madukwe

**Affiliations:** 1 Department of Medical Laboratory Science, University of Jos, Plateau State Nigeria; 2 Meena Histopathology and Cytology Laboratory, Jos Plateau State, Nigeria; 3 Department of Medical Laboratory Science, University of Maiduguri, Borno State, Nigeria; 4 National Hospital Abuja, Nigeria

**Keywords:** Benign hyperplasia, cancer of prostate, co-expression, monoclonal antibodies

## Abstract

**Introduction:**

Diagnosis of prostatic diseases with Immunohistochemistry still faces challenges because of the peculiar histology of the prostate and difference(s) in reactivity of Monoclonal antibodies (MoAb) to benign and malignant changes.

**Methods:**

Thirty (30) archived paraffin embedded tissue samples from primary prostate tumors (15 Benign Prostatic Hyperplasia (BPH) and 15 Cancer of the prostate (CaP)) were sectioned at thickness of 5µm and confirmed as BPH or CaP. Sections from each sample were stained by Immunohistochemistry using the Streptavidin-biotin method and using CK5/6, CK7, CK8,CK20 and Ki67 antibodies (Zymed Antibody products). Appropriate positive and negative controls for each antibody were setup alongside the test slides.

**Results:**

BPH samples were reactive to Ck5/6 (93.3%), Ck7 (80%) and Ck8 (100%). Only 13.3% of BPH samples were reactive to Ki67. The reactivity of Ck5/6, 7, 8 in CaP is a contrast with only 3(20%) of samples positive with Ck5/6, 2(13.3%) positive with Ck7 and 14(93.3%) with Ck8. While reactivity of Ck 8 is similar in BPH and CaP, no reaction was recorded in Ck 20 in both BPH and CaP. Ki67 was only reactive in 2(13.3) of BPH samples and 15(100%) of CaP. Only Ck 8 was expressed in both BPH and CaP. There was co-expression of Ck5/6, 7,8 and Ki67 in13.3%; Ck7and Ki67 in 13.3% in both BPH and CaP.

**Conclusion:**

The various cytokeratins are individually expressed in both BPH and CaP. Ck5/6 and Ck7 are co-expressed and may be used in the diagnosis of BPH, Ck5/6,7,8 and Ki67 are co-expressed in Prostatic adenocarcinoma and squamous cell carcinoma of the prostate while Ck8 and Ki67 are co-expressed and may be used for diagnosis of Prostatic adenocarcinoma alone.

## Introduction

The prostate is the site of two of the most common diseases in elderly men, Benign Prostatic Hyperplasia (BPH) and Prostate cancer (CaP). Both of these conditions are disorders of cell differentiation and cell proliferation [1]. Prostate cancer is the second most frequently diagnosed cancer as well as the sixth leading cause of death in males worldwide [2]. Little is known about the basic biology of cell phenotypes in either normal prostate or prostatic cancers. Phenotypes that are intermediate between those of basal and luminal cells in the heterogeneous prostate epithelial cells have been reported [3]. The prostate gland contains epithelial cells expressing two major phenotypes: luminal and basal cells separated by basement membrane from the stroma. If appropriate makers can be used, characterization of the phenotypes of cells will thus represent a major advantage [4].

The variety of morphologic patterns of different entities of the genitourinary tract can represent a diagnostic dilemma for the pathologist. This is especially true in cases that mimic cancer, in cancer of unknown primary or poorly differentiated tumors in which it is hard to assign histiogenesis needed to plan the correct therapy for the patient [5]. It has also observed that routine staining of prostatic lesions sometimes cause diagnostic dilemma especially in premalignant lesions like atypical adenomatous hyperplasia and prostatic intraepithelial neoplasia (PIN) [6].

The use of Immunohistochemistry in diagnosis is presently widespread because of its sensitivity and specificity. However, diagnosis of prostatic diseases with Immunohistochemistry is still facing challenges because of peculiar histology of the prostate and difference(s) in reactivity to Monoclonal antibodies (MoAb) by benign and malignant changes.

The expression of cytokeratins in prostatic epithelium is varied and is well recognized [7]. It has been reported that Immunohistochemistry offers a better capacity than Haematoxylin and eosin staining alone and its addition to the diagnostic armamentarium for genitourinary pathologic diagnosis has increased the sensitivity and specificity of diagnosis and aided in the selection of optional therapeutic regimens in selected cases [5]. The first study utilizing anti keratin monoclonal antibodies was reported [8]. Others reported immunoreactivity of keratin in basal cell of normal and hyperplastic prostatic epithelium with no staining of adenocarcinoma [9, 10]. Conversely, keratin was identified in one case of prostatic adenocarcinoma using polyclonal anti serum raised against bovine muzzle pre-keratin [11]. It was further reported that keratin immunoreactivity differs in the two epithelial cells of the prostate, probably due to expression of different keratin proteins [12].

Several biological markers have been reported in literature to be good objective markers of progression of different neoplasms. The Ki67 antigen is a useful proliferation marker [13]. The potential of Ki67 antibody for diagnostic purpose was investigated, assessing the proliferation activity in normal prostate tissue, Prostatic Intraepithelial Neoplasia and Prostatic Cancer patients [14]. Cell proliferation is a fundamental aspect of a number of prostatic diseases ranging from hyperplasia to neoplasia [14]. It was reported that cellular proliferation can be studied using Immunohistochemical marker directed against nuclear antigen Ki67 [15].

This study therefore examined the individual expression and the then co-expression of some selected cytokeratins and Ki67 in benign and malignant prostatic biopsies with a view to find out differential, combinations of individual or co-expressed proteins that could make diagnosis more specific and precise.

## Methods

Thirty (30) archived paraffin embedded tissue samples from primary prostate tumors comprising 15 benign prostatic hyperplasia (BPH) and 15 cancer of the prostate (CAP) were sectioned at thickness of 5µm. One section from each sample was stained by the Haematoxylin and eosin method to reconfirm diagnosis of BPH and CaP. Subsequently 2µm sections from each sample were stained by Immunohistochemistry using the Streptavidin-biotin method using the following antibodies CK5/6, CK7, CK8, CK20 and Ki67 (Zymed Antibody products). Negative and positive controls for each antibody were set up alongside the test slides. The positive controls for Ck5/6, 7 and 8 was the normal ureter tissue which are known to express the antigens, while the control for Ck 20 was malignant colonic tissue which expresses the antigen. The control for Ki 67 was a reactive lymphoid hyperplastic tissue. For the negative controls, test tissue sections were used but the antibodies were replaced at the appropriate stage by Tris buffered saline to confirm that all antibodies are reactive and there was no cross reactivity. All sections were dewaxed and hydrated. They were subsequently heated at 1210C for 10minutes in 10mmol/L Citrate buffer (pH 6.0) and the antibodies applied for 45minutes after passing through peroxidise and protein blocks. The secondary biotin-labeled antibody was incubated for 15min at room temperature. The streptavidin labelled streptavidin-biotin amplification method was carried out for 30minutes followed by 3,3’-Diaminobenzidine (DAB) and haematoxylin counter stain were subsequently applied in sequence. Reactivity was observed as brown coloration. Photomicrographs of reactive sections were taken.

The necessary institutional ethical clearance was sought and obtained from Meena Histopathology laboratory.

## Results

**Figure 1:**
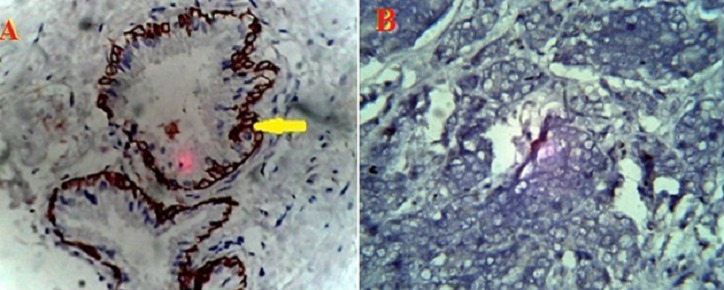
Cytoplasmic Immunohistochemical expression of Ck5/6 (arrow) in prostatic hyperplasia defining the luminal epithelium (A) and no expression in adenocarcinoma (B). (MagnificationX400)

**Figure 2:**
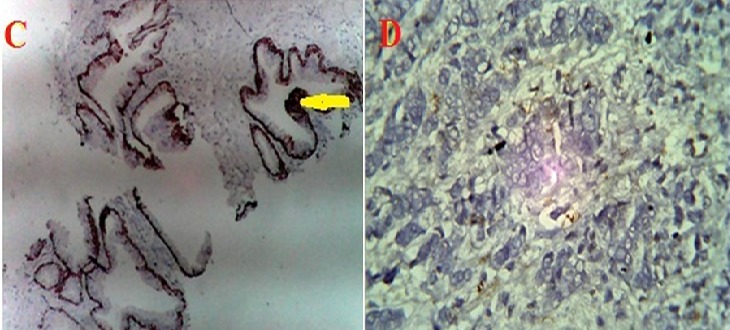
Cytoplasmic Immunohistochemical expression of Ck 7 (arrow) in prostatic hyperplasia defining the luminal epithelium(C). No expression in adenocarcinoma (D). (Magnification X400)

**Figure 3:**
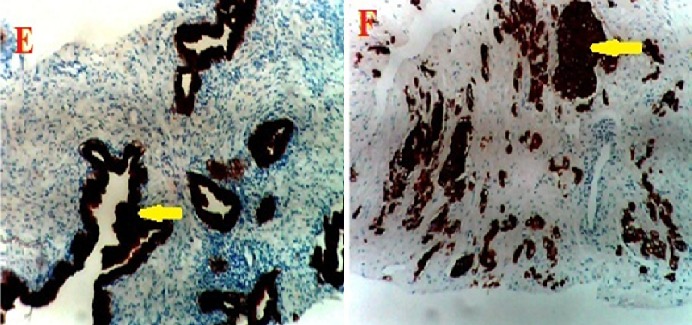
Intense cytoplasmic immunohistochemical expression of Ck 8 in prostatic hyperplasia defining the luminal epithelium(arrow) (E). Histologic section of prostate showing Ck8 immunoreactive atypical acinar proliferation (arrow) in adenocarcinoma (F). (Magnification X400)

**Figure 4:**
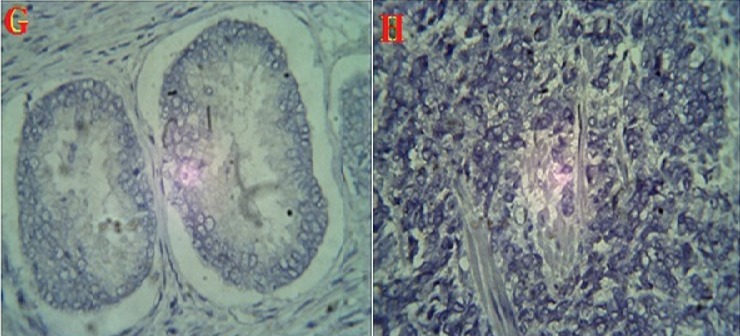
Ck 20 was not expressed in both prostatic hyperplasia (G) and adenocarcinoma (H). (Magnification X400)

**Figure 5:**
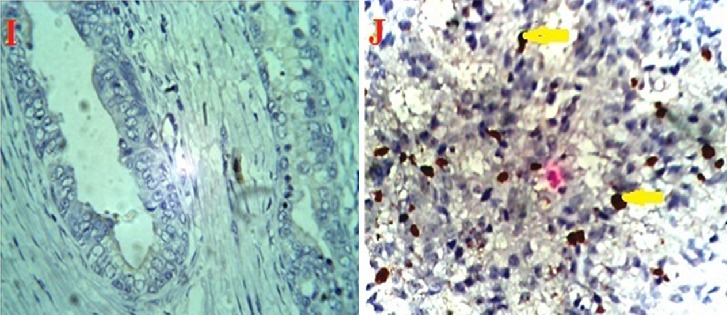
Histologic section of prostate showing no expression of Ki67 in prostatic hyperplasia (I) but immunoreactivity expression in adenocarcinoma(J). (Magnification X400)

The percentage of cases that expressed each of the monoclonal antibodies (MoAb) in BPH and CaP are presented in Table 1. Ck5/6 (Figure 1) was expressed in 93.3% of BPH samples and 20% of CaP samples. Ck 7 (Figure 2) was expressed in 80% of BPH samples and 13.3% of CaP cases. Ck8 (Figure 3) Ck 8 was expressed in virtually all BPH and CaP samples (100% and 93.3% respectively). There was no expression of Ck20 (Figure 4) in both BPH and CaP. Ki67 was expressed in 100% of CaP and 13.3 of BPH (Figure 5).

**Table 1:** Expression MoAb in benign prostate hyperplasia and prostate cancer (%)

Prostatic tumors	Ck5/6	Ck7	Ck8	Ck20	Ki67
**CaP**	20	13.3	93.3	-	100
**BPH**	93.3	80	100	-	13.3

The co-expression of cytokeratins and Ki67 in BPH and CaP are represented in Table 2. The percentages of samples that co-expressed Ck 5/6, 7 and 8 are 73.3% and 13.3% for BPH and Cap respectively. Co-expression of Ck 5/6,7,8 and Ki67 was obtained in 13.3% of both BPH and CaP samples. The co-expression of Ck 5/6 and Ki67; Ck& and Ki67; Ck 8 and Ki67 are 13.3/20; 13.3/13.3 and 13.3/93.3 percents in BPH and CaP respectively.

**Table 2:** Co-expression of cytokeratins and Ki67 in benign prostate hyperplasia and prostate cancer (%)

Ck5/6,Ck7 &Ck8	Ck5/6,Ck7, Ck8&ki67	Ck5/6,ki67	Ck7/ki67	Ck8/ki67
13.3	13.3	20	13.3	93.3
73.3	13.3	13.3	13.3	13.3

The number of monoclonal antibodies in the panel used reactive in BPH and CaP are presented in Table 3. The highest numbers of MoAb reactive in majority of BPH and CaP samples are 2 and 3. Two (2) MoAb are expressed in over 70% of CaP samples and 28% of BPH while 3MoAb are expressed in about 60% of BPH samples and less than 10% of CaP samples. Expression of 4 Moab was obtained in a little higher than 10% of both BPH and CaP samples.

**Table 3:** Number of MoAb reactive in benign prostate hyperplasia and prostate cancer (%)

Number of monoclonal antibodies			
1	2	3	4
6.7	73.3	6.7	13.3
-	26.7	60	13.3

## Discussion

The co-expression and individual expression of CK5/6, Ck7, Ck8, Ck20 and Ki67 in BPH and CaP was examined. This information intends to make distinction between benign and malignant diseases of the prostate clearer. Our study shows that majority of BPH samples were reactive to Ck5/6 (93.3%), Ck7 (80%) and Ck8 (100%) (Table 1) and is agreeable with previous research [16]. Only 13.3% of samples were reactive to Ki67. This is understandable because BPH is benign and Ki 67 is known to be a proliferative MoAb and is also consistent with previous reports [10, 17]. The observation of cytokeratins (Ck5/6and7) in a few CaP samples was noted. It was however, observed that this occurred only in poorly differentiated squamous cell carcinoma. The only case that was not reactive with the cytokeratins is the well differentiated squamous cell carcinoma. Other researchers [18, 19] similarly reported that cytokeratins are differentially expressed in normal as well as abnormal prostate epithelia. This is significant when co-expression of monoclonal antibodies is used for differential diagnosis.

The reactivity of Ck5/6, 7, 8 in CaP is a contrast with only 3 (20%) of samples positive with Ck5/6, 2(13.3%) positive with Ck7, but 14(93.3%) with Ck8. While reactivity of Ck 8 is similar in BPH and CaP, no reaction was recorded in Ck 20 in both BPH and CaP. Ck 20 has been reported [20] to be absent in the prostate. The presence of CK8 in both BPH and CaP is said to be normal to the prostate stem cells therefore cannot be used as a diagnostic biomarker [21] Ki67 was only reactive in 2(13.3) of BPH samples and 15(100%) of CaP. The distinction between the two is that reactivity in BPH was weak while in CaP reactions were strong. This finding is consistent with that of previous studies and has been reported as a proliferation marker with demonstrated usefulness in many neoplasms including the prostate. It is used in determining their progression [22–24].

Co-expression of MoAb in BPH and CaP is represented in Figure 2 and Figure 3. Only Ck 8 was expressed in both BPH and CaP. What distinguishes BPH and CaP is the fact that most BPH were not reactive with Ki67 and therefore not co-expressed with Ck 8 in contrast to CaP in which Ck8 and Ki67 are co-expressed in 100% of samples. The same cannot be said of the other Ck5/6, 7, 20 and Ki67. There is however various degrees of co-expression although in less than 20% of the samples. These include the co-expression of Ck5/6, 7,8 and Ki67 in13.3%; Ck7and Ki67 in 13.3% that cuts across BPH and CaP. It was also, observed that these coincided with samples that had a diagnosis of squamous cell carcinoma. This is significant in the differential diagnosis of squamous cell carcinoma of the prostate and Prostatic Adenocarcinoma.

## Conclusion

Definitive diagnosis of prostatic tumors using individual expression and co-expression of monoclonal antibodies is critical to the selection of options for therapeutic regimens and therefore the information obtained can be used although to a limited extent to make diagnosis more accurate. Consequently, the pathologist needs to be knowledgeable in the use of co-expressed antibodies to make decisions in diagnosis.
